# Genetic, Phenotypic, and Commercial Characterization of an Almond Collection from Sardinia

**DOI:** 10.3390/plants7040086

**Published:** 2018-10-15

**Authors:** Emma Rapposelli, Maria Pia Rigoldi, Daniela Satta, Donatella Delpiano, Sara Secci, Andrea Porceddu

**Affiliations:** 1 Servizio per la Ricerca in Arboricoltura, Agenzia per la Ricerca in Agricoltura della Regione Autonoma della Sardegna (AGRIS Sardegna), Via Demartini 244, 07100 Sassari, Italy; emmarapposelli@gmail.com (E.R.); dsatta@agrisricerca.it (D.S.); sarasecci@gmail.com (S.S.); 2 Servizio Ricerca nelle Filiere Olivicolo-Olearia e Viti-Enologica, Agenzia per la Ricerca in Agricoltura della Regione Autonoma della Sardegna (AGRIS Sardegna), Strada Statale 196 Villasor-Villacidro, Km. 14.600, 09034 Villasor, Italy; dodelpiano@agrisricerca.it; 3 Agronomia, Coltivazioni Erbacee e Genetica, Dipartimento di Agraria, Università degli Studi di Sassari, via Enrico De Nicola, 07100 Sassari, Italy; aporceddu@uniss.it

**Keywords:** almond, kernel quality, oil composition, flowering time

## Abstract

Background: Recent nutritional and medical studies have associated the regular consumption of almonds with a wide range of health benefits. As a consequence, kernel quality has become an important goal for breeding, considering not only the chemical composition conferring a specific organoleptic quality but also physical traits related to industrial processing. Methods: We characterized an almond collection from Sardinia through analysis of 13 morpho-physiological traits and eight essential oil profiles. The genetic structure of the collection was studied by analyzing the polymorphism of 11 simple sequence repeats (SSR). Results: Both commercial and phenotypic traits showed wide ranges of variation. Most genotypes were early flowering with low yield potential. Several genotypes showed moderate to high yield and very interesting oil compositions of kernels. Based on 11 SSR profiles and Bayesian clustering, the Sardinian cultivars were assigned to groups which were differentiated for several agronomic and commercial traits. Conclusions: Several cultivars showed a high kernel oil content and high oleic to linoleic content ratio. Based on morphological traits, we propose that some of the analyzed cultivars could be interesting for industrial applications. Finally, we highlight the importance of characterizing early blooming cultivars for sites which are experiencing a rise in mean temperatures due to the effects of global climate changes.

## 1. Introduction

Almond (*Prunus amygdalus* Batsch, syn. *Prunus dulcis* Miller D.A. Webb, syn. *Amygdalus communis* L.) is an important nut crop that is cultivated from the desert areas of Western China to the Mediterranean basin [1]. The Greeks introduced almond cultivation to Italy in the fifth century B.C. [2]. Almond cultivation is now prevalently carried out in Southern Italy, and particularly in Apulia (27,500 t in 2017) and Sicily (51,300 t produced in 2017; Italian National Statistics Institute, 2017 data). In these areas, a small number of commercial cultivars provides most of the almond production, including *Genco*, *Lauranne*, *Moncayo*, *Tuono*, *Ferragnés*, and *Filippo Ceo*. Small-scale production based on local germplasm is prevalent in other Italian regions, such as Sardinia (4444 t produced in 2017), Calabria (735 t in 2017), Abruzzo (24 t in 2017), and Basilicata (412 t in 2017).

The quality of almond crop relates to the commercial, nutritional, and organoleptic aspects of the nut and kernel [3,4,5]. The confectionery and cosmetic industries require kernels with high chemical quality; e.g., kernels with high percentages of oil are suitable for production of nougat or for extraction of their oil for cosmetics and pharmaceuticals [6].

Both epidemiological and clinical studies have indicated that almond consumption is associated with reduced cardiovascular risk and favorable plasma lipid profiles [7,8]. More interestingly, whole almond kernels and almond oil do not differ significantly in their beneficial effects, which indicates that the favorable effects of almond nut consumption are mediated through the components of their oil fraction [9]. As well as providing beneficial nutritional value, the lipid content and composition of almonds are also important for oil stability, as the fatty acid components differ in their vulnerability to oxidation. Polyunsaturated fatty acids are more susceptible to oxidation than monounsaturated fatty acids, thus, Kester et al. [10] proposed an index of resistance to oil rancidity based on the ratio of the oleic to linoleic acid contents. The resistance to oil rancidity also depends on the presence of natural antioxidants, such as the tocopherols [11,12]. Such natural antioxidants can allow longer kernel storage times, as they protect against fat oxidation [13,14]. The tocopherols are also important for human health; indeed, α-tocopherol is also known as vitamin E, and almonds have the highest tocopherol content seen for nuts [15].

Interest in the characterization of almond quality has recently increased following the demonstration that although the composition of almond oil depends primarily on the genotype, it is also defined by the environmental conditions prevalent during the growing season, location, and climate [16,17,18]. These data are in agreement with studies that have indicated that agronomically obsolete almond cultivars and local genotypes can have good oil quality and other traits that are of commercial interest [19,20]. The identification of cultivars with particular qualitative characteristics, although with reduced productive potential, might also stimulate their cultivation for purposes such as cosmetic/pharmaceutical and characterization of typical foods.

The objectives of this study were thus threefold: (i) to study the genetic and phenotypic structure of a Sardinian almond collection; (ii) to define their quality traits; and (iii) to investigate the relationships between their genetic clustering and phenotypic and oil traits.

## 2. Results

The collection analyzed in this study included 38 almond genotypes cultivated in Sardinia and seven reference cultivars (Table 1), representing several almond cultivation regions. *Non Pareil* and *Ne Plus Ultra* are two reference cultivars mainly cultivated in California [21]. *Tuono* and *Genco* are among the most widespread cultivars in Italy [22]. *Troito A* and *Troito B* are *Tuono*-related cultivars which are cultivated in Greece [23]. Finally, *Picantili* is a reference cultivar that originated in Russia [24].

### 2.1. Flowering-Related Traits

The flowering-related parameters showed a wide range of variation among Sardinian cultivars. As reported in Table 1, the earliest flowering cultivar, *Stampasaccusu*, started flowering at 18.67 days (after 1 January), while the last, *Sunda N.*, flowered at 55.67 days (*p* < 0.05). The mean flowering date across all of the Sardinian cultivars was 38.23 days. Note that the earliest commercial cultivar, *Ne Plus Ultra*, started flowering at 36 days while the latest two, *Troito A* and *Genco*, flowered at 55 and 67 days, respectively (*p* < 0.05). See Supplemental Table S1 for details on observed standard deviations.

The analysis of variance showed that both the cultivar and the year factors were discriminated for blooming timing (see Table 2).

### 2.2. Nut- and Kernel-Related Traits

Analysis of variance showed that the genotype significantly affected all investigated nut- and kernel-related traits (see Table 2). The year showed a significant effect on kernel weight and shelling percentage, while no significant effect was observed for the other traits.

Nut weight showed high variation (Table 3), from a minimum of 1.33 g for *Non Pareil* to a maximum of 7.47 g for the Sardinian cultivar *Basibi* (*p* < 0.05: see Table 3). The lightest nuts among the Sardinian cultivars were those of *De Efisi Sinzoba* (2.29 g). The mean nut weight across all of these Sardinian cultivars was 5.57 g. Kernel weight varied across a 2.2-fold range (*p* < 0.05), with the heaviest being *Bianca* (1.84 g) and the lightest being *Nuxedda* (0.84 g). The percentage of double kernels was highly variable too, from virtually no double kernels for *Riu Loi*, *Nuxedda*, *Farrau*, and *De Mrasciai*, up to 48.33% for *Pitichedda* (*p* < 0.05; Table 3). Also, the reference cultivars showed a wide range of variation for this trait, ranging from 2% in the cultivar *Genco* up to 30.6% of *Tuono* (*p* < 0.05). Failed kernels were virtually absent for *De Mrasciai*, *Is Stumbus*, *Pitichedda*, and *Vavani Perra*, again in striking contrast to the 13.00% failed kernels of the reference cultivar *Non Pareil* (*p* < 0.05; Table 3).

*Nuxedda* had the shortest nuts (2.43 cm), while *Ne Plus Ultra* had the longest (4.05 cm; *p* < 0.05), with *Malissa Tunda* being the longest among the Sardinian cultivars (3.73 cm; *p* < 0.05). *Niedda I* had the widest nuts, with a mean width of 3.34 cm, as compared to the narrowest of 1.80 cm for *Non Pareil* (*p* < 0.05; see Table 3).

### 2.3. Kernel and Oil Content Composition

Analysis of variance showed that both genotype and year significantly affected kernel oil composition (Table 4). The interaction cultivar by year was significant (*p* < 0.05) for all oil components and also for the total oil content of kernels. The factor year explained more than cultivar and interaction for all oil components but not for total oil content, which was mainly accounted for by cultivar (see Table 4).

The kernel oil content of Sardinian cultivars varied from the mean 64.47% of *Ibba* to that of 52.03% of *Malissa Tunda* (*p* < 0.05; Table 5). The fatty acid composition of the kernels was also highly variable across the cultivars. By way of example, the maximum palmitic acid was seen for *Farrau* at 7.38%, with the minimum for *Vargiu* at 5.56% (*p* < 0.05).

Palmitoleic acid showed the extremes of 0.77% and 0.38% for *Ciatta Inglese* and *Malissa Tunda*, respectively (*p* < 0.05). The stearic acid content varied from 2.42% for *Cossu* to 1.36% for *Malissa Tunda* (*p* < 0.05). *De Mrasciai* had the highest oleic acid content (78.29%) and the lowest linoleic acid content (13.40%), while at the opposite extreme, *De Efisi Sinzoba* had the lowest oleic acid content (68.64%) and the highest linoleic content (22.22%; *p* < 0.05).

α-Tocopherol (a form of Vitamin E) is an important molecule with antioxidant activity that has beneficial effects for human health. *Farci* had the highest α-tocopherol relative content, 589.41 mg/kg, while *Emilio 91* had the lowest at 301.28 mg/kg, as shown in Table 5 (*p* < 0.05). By comparison, the α-tocopherol contents of the reference cultivars *Tuono* and *Genco* were 540.84 mg/kg and 226.90 mg/kg, respectively (*p* < 0.05).

**Figure 1:**
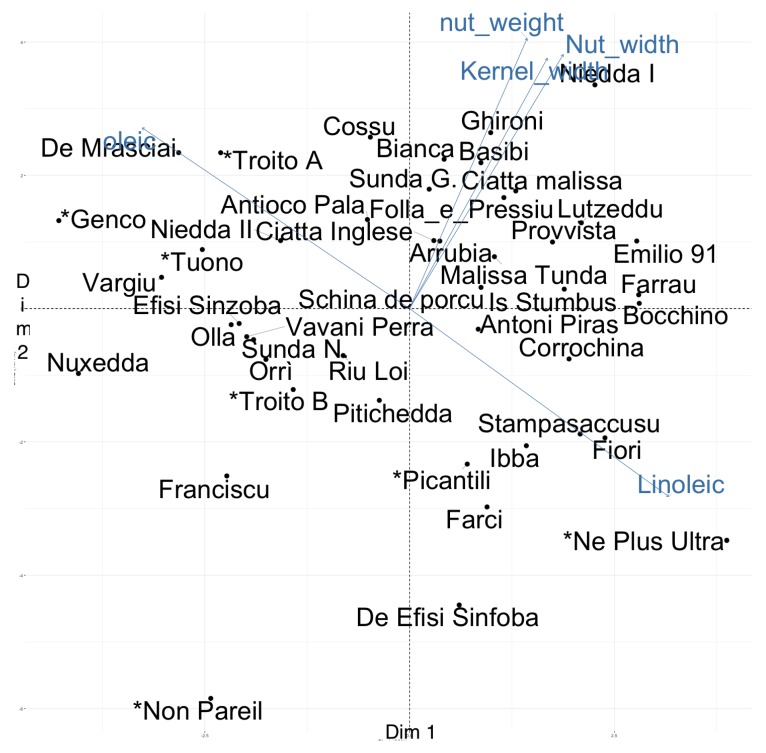
Biplots of individuals and variables in the principal component analysis (PCA) components 1 and 2. Only the five variables giving the highest contributions are shown. The names of reference cultivars are preceeded by an asterisk.

Principal component analysis (PCA) was used to summarize the relationships between the cultivars based on these phenotypic and chemical traits. The first two PCA components accounted for 39.06% of the total variance. The first component accounted for 19.98% of the variance and showed high contributions for both phenotypic and chemical traits (Figure 1). The linoleic acid and oleic acid contents and, to a lesser extent, kernel length and nut length were the major contributions to the first component.

**Figure 2:**
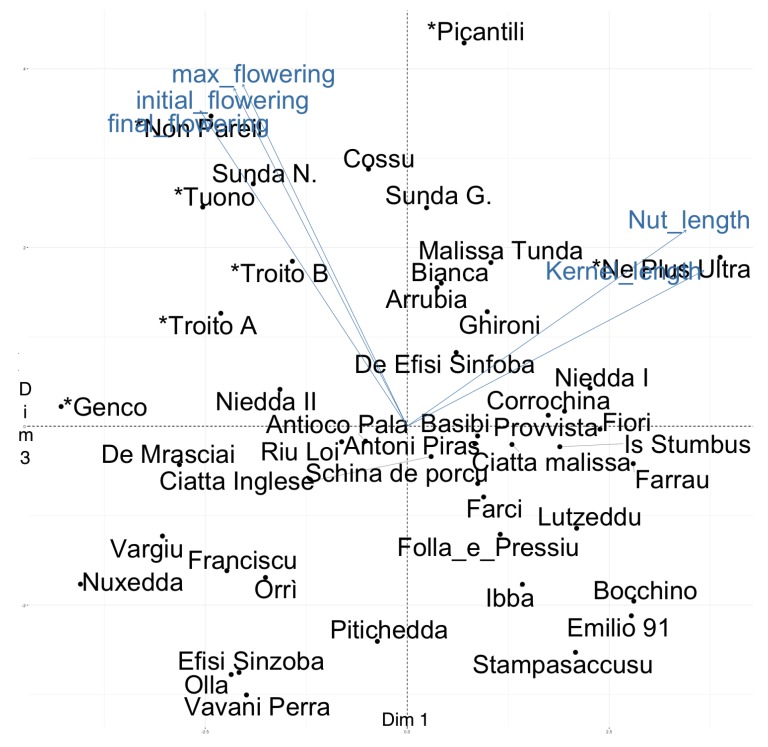
Biplots of individuals and variables in the PCA components 1 and 3. Only the five variables giving the highest contributions are shown. The names of reference cultivars are preceeded by an asterisk.

Most of the reference cultivars, such as *Genco*, *Non Pareil*, *Tuono*, *Troito A*, and *Troito B*, are all positioned on the left side of the PCA 1–2 biplot (Figure 1), mainly owing to their high oleic relative content. We found several Sardinian cultivars, such as *Nuxedda*, *De Mrasciai*, and *Vargiu*, which, due to the high compositional quality of their kernel oils, were positioned in close proximity to these reference cultivars along the first PCA. In particular, the cultivar *Vargiu* showed one of the highest oleic to linoleic content ratios and narrow nuts and kernels (see also Table 3 and Supplemental Table S1). It is noteworthy that the group composed of the cultivars *Olla*, *Efisi Sinzoba*, *Vavani Perra*, *Sunda N.*, and *Orri* showed scores similar to that of the reference cultivar *Troito B* along PCA 1 and 2 (Figure 1). Since the flowering-related traits showed the highest contributions to the third PCA component, we summarized the combination of phenological and commercial traits by visualizing the scores in the third PCA along with those in first PCA component (Figure 2). The second component in the PCA accounted for 19.08% of the total variation, with high contributions for traits related to fruit characteristics: nut weight, nut width, and kernel width and, to a lesser extent, shelling percentage.

For a complete view of the loadings on the PCA axes, see Supplemental Table S2. Among the early flowering cultivars, *Efisi Sinzoba*, *Olla*, and *Vavani Perra* showed a high rancidity ratio (Supplemental Table S1). Again, we highlight the cultivar *De Mrasciai*, which showed a high quality of kernel oils and a late flowering habitus.

### 2.4. Genetic and Phenotypic Similarities among Cultivars

**Figure 3:**
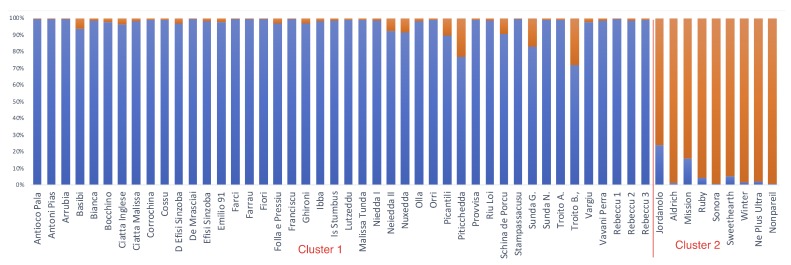
Most probable genetic structure of Sardinian and reference national and international genotypes, as revealed by STRUCTURE analysis. Bars represent individuals, and coefficients of membership (Q) to specific clusters are reported with different colors. Cluster assignment was based on a membership threshold set at >0.75. CL1 (blue), CL2 (red).

In a previous work, we demonstrated that based on the genotype at 11 simple sequence repeat (SSR) loci, the Sardinian almond cultivars could be distinguished from most cultivars from Apulia but not from USA reference cultivars [25]. Here, we deepened the analysis on the relationship between the Sardinian and USA reference cultivars. Note that to gather a higher resolution, additional USA varieties and three bitter almond Sardinian cultivars were included in the analyses (see Supplemental file S1 for details on these cultivars). Model-based clustering of the SSR data identified two clusters as the most probable dataset partition [25]; (see Supplemental file S1 for best K determination): cluster 1 (CL 1), which included all the Sardinian cultivars plus the reference cultivars *Picantili*, *Troito A*, and *Troito B*; and cluster 2 (CL 2), which included all the USA reference cultivars (see Figure 3).

**Figure 4:**
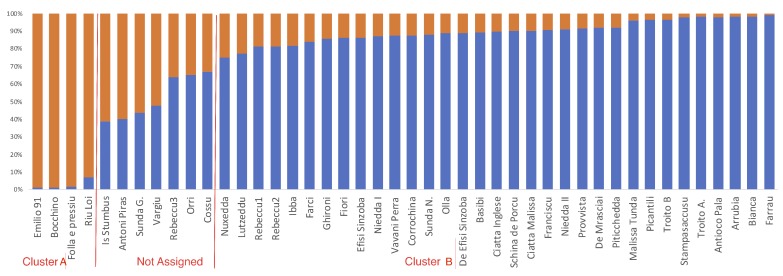
Most probable partition of cluster 1, as revealed by STRUCTURE analysis. Bars represent individuals, and coefficients of membership (Q) to specific clusters are reported with different colors. Cluster assignment was based on a membership threshold set at >0.75.

Next, we investigated the genetic structure within cluster 1. Structure analysis identified the most probable partition as two subgroups: CL-A and CL-B (see Supplemental file S1 for details on best K determination; Figure 4).

The cultivars *Is Stumbus*, *Antoni Piras*, *Sunda G.*, *Vargiu*, *Rebeccu 3*, *Orri*, and *Cossu* were not assigned to any group, as none of their memberships reached the threshold for assignment (Q > 0.75).

As the first step in assessing the relationships between the genetic and phenotypic diversity, we investigated the association between the collection partition based on the genetic data, and the morphological and chemical traits. The three Rebeccu cultivars were not considered for these analyses because they produce bitter kernels. Cluster CL-*A* was the first to flower and to achieve maximum flowering (see Table 6). This cluster was distinguished from the other cluster also for the width of kernels (see Table 6). Of note, cluster A also showed an average α-tocopherol content below 350 mg/kg, while the average α-tocopherol of cluster 2 was 420 mg/kg. The average stearic acid content of cluster A was 1.98 compared to 1.81 of cluster B.

## 3. Discussion

In the present study, we report the characterization of a collection of 38 Sardinian almond local cultivars. Most of these genotypes were early blooming and showed wide ranges of variation for several quality-related traits. The kernel oil content ranged from 52.3% in the cultivar *Malissa Tunda* up to 64.47% in the cultivar *Ibba*. Such a range of variability is in line with data reported for other collections of commercial or local almond genotypes. For example, Kodad et al. [26] reported comparable ranges of variability in the kernels of 73 almond cultivars typical of 10 almond-producing countries and grown at the CITA Institute. Similar ranges of variability were detected for almond cultivars grown in Egypt [27], Greece [28], India [29,30], Iran [31], and Italy [32,33]. Higher ranges of variability were reported in studies which considered breeding trials, such as Turkish selections (25–61%) [34,35] or Afghan [36] and Spanish selections [6,12] (43–63% and 40–67%, respectively). Interestingly, lower ranges of variability were reported for a collection of California almond cultivar and breeding selections, resulting from peach gene introgressions, and in a comparative study between Californian and European cultivars cultivated in Spain [16].

High variability has been previously recorded also for individual oil components. Yada et al. [4] reported that in commercial almond cultivars grown in various regions, oleic and linoleic content accounts for about 90% of the total lipids. Noteworthy is that all the Sardinian genotypes analyzed in this study showed a total content of oleic and linoleic acid higher than 90% of the total lipids. A high oleic acid content is desirable from both the quality and stability points of view, as it increases the nutritional value and the stability against rancidity [10]. The oleic acid content ranged from 68.64% in the cultivar *de Efisi Sinzoba* up to 78.29% in the cultivar *De Mrasciai*. Notably, the highest relative oleic acid content in the Kodad study was 78.4% for the *Yosemite* kernels. The kernels of the Californian cultivar *Ne Plus Ultra* showed 66.53% oleic acid content, the lowest value measured in this study.

It is important to underline that fatty acid content and the composition of kernels can be significantly influenced by the environment. Hence, the comparison of data obtained from different years or experimental sites should be always considered with caution. Indeed, we have found that the factor year significantly affected several morphological and commercial traits. Several studies have demonstrated that the heritability of oil content and composition of almond kernels is rather high, and thus, the kernel oil content and composition is expected to be substantially determined by the genetic background of the almond cultivars [4]. We found that the cultivar was the main determinant of the total oil content of the kernels, while the year was the main factor for most oil components. It is very important to underline that also the interaction cultivar x year was of moderate entity, though significant, for all oil components analyzed, a finding in substantial agreement with data reported by other studies [19].

The tocopherols in kernel protect polyunsaturated fatty acids against peroxidation [37]. They also have protective roles in human health due to hypocholesterolemic, anticancer, and neuroprotective properties [37]. We found that the Sardinian cultivars showed a wide variation of α-tocopherol content in kernels, ranging from a minimum of 301.28 mg/kg of oil of the cultivar *Emilio 91* to 589.41 mg/kg of oil of the cultivar *Farci*. These ranges are in agreement with data reported for Spanish (335–551.7 mg/kg) [15] and Italian cultivars [38] (350–471 mg/kg) but slightly lower than those reported for Moroccan and Californian cultivars (300.9–646 mg/kg) [19,39]. However, it is important to underline that tocopherol accumulation in almond kernel is significantly affected by drought stress and temperature during kernel maturation [37]. The highest tocopherol concentrations (646 mg/kg) were found when the almond development coincided with spring and summer, with a warmer mean temperature in studies conducted in Northwestern Argentina or Afghanistan [39,40]. Therefore, it is possible that the early blooming Sardinian cultivars may have the potential to accumulate higher levels of tocopherol if grown in environments with higher mean temperatures during kernel development.

Kernel size and weight have been considered important targets in almond breeding. Efforts have been made to select genotypes with an average kernel size greater than 1 g. Notably, all but two Sardinian genotypes showed an average kernel weight heavier than 1 g. The cultivar *Bianca* produced the heaviest kernels (1.84 g) and the cultivar *Nuxedda* the lightest (0.84 g). Maestri et al. [41] considered kernel weight in a selection of traditional cultivars and native almond genetic resources from Argentina, reporting ranges from 0.86 to 1.56 g. The heaviest kernels were produced by the cultivar *Caceres* (1.56 g), showing a weight remarkably greater than that observed for the cultivars *Guara* (0.90 g), *Non Pareil* (0.86 g), etc. Other studies reporting on Spanish, Italian, North American, as well as from various Turkish almond genotypes reported values lower than those registered for the best Sardinian genotypes.

The availability of molecular data allowed us to assign the Sardinian cultivars to genetic clusters and to analyze the associations between the identified clusters and the average agronomic and commercial performances. The Sardinian cultivars were clearly distinguished from USA cultivars but not from some commercial cultivars, such as *Picantili* and the cultivars *Troito A* and *Troito B*. The separation of cultivars based on geographic origin has been already reported by Fernandez et al. [42]. Based on a model-based Bayesian clustering approach, we assigned the analyzed Sardinian genotypes to two clusters which were differentiated for agronomic and commercial traits. The genotypes belonging to CL-A were early flowering and also showed a lower α-tocopherol content. Based on the combination of several traits, we indicate some Sardinian cultivars which, in our opinion, may have the potential for industrial processing. Specialized uses, such as the inclusion in chocolate bars, require small kernels. For example, two highly considered cultivars by the chocolate industry—the Spanish cultivar *Felisia* and the American cultivar *Milow*—have kernel weights of 0.85 and 0.82 g, respectively. The cultivar *Nuxedda* showed a kernel weight of 0.84 g and a rancidity index of 4.75, with oleic acid representing more than 75% of the total kernel oil. *Olla* is another interesting cultivar, with 75.52% oleic acid and 15.61% linoleic acid. Both of these cultivars have low productivity but, due to their high oil quality, they deserve attention for specific uses or as parents for breeding programs. Noteworthy is that these two cultivars have similar scores along the first and second PCA axes which are correlated with the morphological and commercial quality of the kernels. Oblong-shaped kernels are highly desirable for sliced or slivered products, as more uniform slices can be obtained from oblong kernels. Several cultivars with long kernels, such as *Ciatta Inglese* (2.42 cm), showed high oleic to linoleic acid content ratios, thus associating a desirable kernel shape to high kernel oil quality. In agreement with other reports, we have shown that early flowering cultivars have, on average, reduced productivity. Among the possible causes subtending such a behavior, we underline the importance of frost damage during flowering. We identified some cultivars, such as *Basibi*, that showed good kernel yields (0.93 kg/plant). These values are comparable to those observed for the reference cultivar *Ne Plus Ultra* and lower than those for the reference cultivars *Genco* and *Tuono*. Noteworthy is that *Basibi* flowered 14 days before *Non Pareil* and 20 and 18 days before *Genco* and *Tuono*, respectively. Thus, these Sardinian genotypes may be considered of some interest for breeding programs employing early flowering germplasm or for extending almond cultivation in environments which allow short endodormancy periods [43,44].

In conclusion, we report a local almond collection showing a high range of variability for several agronomical and commercial traits. Most of these genotypes were early blooming and showed exceptional properties in terms of kernel and oil quality. We propose that these genotypes should be taken into consideration as interesting resources for breeding programs or for extending almond cultivation to sites which are predicted to experience a rise of mean temperatures following the effects of global climate changes. Indeed, as reported by Prudencio et al. [44], there is a risk in growing late-flowering cultivars in warm areas, since the chilling temperatures needed to break dormancy may not be reached, and this could affect dormancy breaking, as well as the quantity and quality of production.

## 4. Materials and Methods

### 4.1. Plant Material

Thirty-eight sweet almond genotypes cultivated in Sardinia and seven reference almond cultivars cultivated worldwide were included in this study (Table 1). These were obtained from the collection maintained by the Sardinian Research Agency (Agenzia per la Ricerca in Agricoltura della Regione Autonoma della Sardegna), Uta, Italy. The plants were grafted into the GF677 rootstock and planted in soil during winter 1989. The trellis system is multi-conical goblet and the plants are 6 × 6 m spaced. The soil is sandy-clay (42% sand) with pH 7.4. The field is cover cropped; weeds along the row are controlled by herbicide application (Roundup). Irrigation on the rows is done by a drip irrigation system from late June to September, depending on the weather conditions.

### 4.2. Phenotypic Traits

The fruit and phenological traits were recorded during 2011–2013 from samples harvested from three plants (replicates) of each almond genotype. For each genotype, 15 fruits from three replicate plants were considered. The samples from the same genotype were pooled together before morphological determinations. Thus, Sardinian and reference cultivars were analyzed according to the almond descriptors developed by the International Plant Genetic Resources Institute (now known as Bioversity International; http://www.bioversityinternational.org/), with some minor modifications [45]. The flowering period was characterized by three parameters: the initial, maximum, and final flowering dates (all as days from 1 January; [45]). The three parameters were detected on 1-year branches (one for each cardinal point) and calculated as 5% opened flowers (initial flowering), 50% opened flowers (max flowering), and 50% of flowers with fallen petals (final flowering). The productive traits were nut and kernel weight (g), kernel percentage, and percentage of nuts without a kernel. The morphological fruit traits were percentage of double kernel, nut length (cm) and width (cm), and kernel length (cm) and width (cm).

### 4.3. Oil Traits

The fruits of each genotype were collected over the three consecutive seasons (i.e., in September 2011, 2012, 2013). The kernels were peeled and then ground in a coffee mill. The flour from each replicate was sieved through an 18-mesh (1 mm diameter) sieve. The dry matter content (dry weight) was calculated for 5 g aliquots of kernel flour after oven-drying overnight at 105 °C. The kernel oil content was expressed as the percentage of the kernel dry weight. The following fatty acids were determined as percentages of total oil content: palmitic and stearic acids (i.e., saturated fatty acids), oleic and palmitoleic acids (i.e., monounsaturated fatty acids), linoleic and α-linolenic acids (i.e., polyunsaturated fatty acids). The α-tocopherol content was also determined (mg/kg oil). The oleic acid to linoleic acid ratios were calculated to infer how prone each cultivar was to onset of rancidity, and thus as indicative of the potential length of storage [10]. The oil was extracted from another 5 g aliquot of each kernel flour through treatment with 80–100 mL petroleum ether (30–50 °C) for 4 h in a Soxhlet extraction apparatus. The petroleum extracts containing the lipids were distilled in a rotary evaporator at 40 °C. Finally, the lipid weight was determined after evaporation of the residual ether under a flow of N2 gas. The total lipid percentages were calculated according to the dry matter determined for the (separately determined) 5 g aliquot of flour. The fatty acids in the oil samples were converted to their corresponding methyl esters. For this, 0.5 g oil was dissolved in 6 mL hexane, and 250 μL 2 N KOH in methanol was added. After moderate shaking, the sample was centrifuged at 2000 gravity for 10 min. The supernatant was transferred to a glass vial for gas chromatography analysis (GC 680; Clarus, Perkin-Elmer Corp, Norwalk, Connecticut, USA) using a 2380 column (Supelco, Pennsylvania, USA; 60 × 0.25 mm i.d.; 0.2 μm film thickness) with a flame ionization detector. The injection volume was 0.5 μL, and helium was used as the carrier gas (flow rate of 0.37 mL/min). The injector and detector temperatures were both set at 220 °C. The initial column temperature was set at 185 °C for 25 min. The oven temperature was then increased to 200 °C with a 10 °C/min ramp, and maintained at 200 °C for 10 min. This was then increased to 220 °C with a 10 °C/min ramp, and maintained at 220 °C for 20 min. The total run time was 58.5 min. The identification of the fatty acid methyl esters was achieved by comparison with the relative retention times in reference samples of each methyl ester standard (Sigma-Aldrich, St. Louis, MO, USA). A Total-Chrome Work Station was used for the data processing.

### 4.4. α-Tocopherol Determination

Samples of 0.1 g oil were dissolved in 1.9 mL acetone, shaken, and filtered through 0.22–4 μm syringe cellulose filters. An aliquot of 20 μL of this solution was injected onto the HPLC system (Waters, Milford, MA, USA), which was equipped with a pump unit (600 Controller; Waters) and an auto-sampler (717 plus; Waters). The chromatography column (Spherisorb ODS2; 250 × 4.6 × 5 μm) was kept at 25 °C, with a pre-column used (Phenomenex cartridge, Torrance, CA, USA, C18 AJO-4287). The mobile phase was acetonitrile and methanol (1:1; *v*/*v*) at a flow rate of 1 mL/min. α-Tocopherol was detected using a photodiode array detector (996; Waters) at a wavelength of 295 nm, using a run time of 18 min. An Enpower 2 Work Station was used for the data processing. The α-tocopherol concentrations were initially in mg/L based on the calibration curve, with α-tocopherol (Sigma-Aldrich) as the external standard. From the oil weight in the 2 mL sample (see above), the α-tocopherol was finally expressed as mg per kg oil (mg/kg).

### 4.5. DNA Extraction and SSR Genotyping

Total genomic DNA was extracted from the powdered leaf of samples using a *GeneElute*^TM^ Plant Genomic DNA Miniprep kit (Sigma-Aldrich). Eleven SSRs were chosen based on chromosome position and amplification quality (see Table S3 for details on primer sequences and annealing temperatures).

Each 25 μL PCR reaction contained 1X PCR buffer (InVitrogen, Carlsbad, CA, USA), 1.5 mM MgCl2, 0.2 mM dNTP, 0.2 μM of each primer (the forward primer was labeled with 6-FAM), 60 ng genomic DNA, and 0.5 U recombinant Taq polymerase (InVitrogen, Carlsband, CA, USA).

The thermal cycling program for UDP and CCPT SSR was composed of: 5 min at 95 °C, followed by 35 cycles of 45 s at 94 °C, 45 s at the temperature of annealing (see Table S3), and 45 s at 72 °C. The thermal program was closed by a final step of 8 min at 72 °C. For BBCT SSR, the initial extension was for 60 s at 94 °C, the annealing step was 45 s at 58 °C, and 2 min at 72 °C was for extension. The amplicons were separated using an ABI PRISM 310 Genetic Analyzer (Applied Biosystems, Foster city, CA, USA) to estimate fragment lengths based on the migration of Genescan^TM^–500LIZ^TM^ size standards.

### 4.6. Genetic and Statistical Analyses

The SSR alleles chosen here were the same as those used by Rigoldi et al. [25]. The genetic relationships among the genotypes were analyzed using a model based on a Bayesian clustering approach, as implemented in the STRUCTURE 2.2 software [46]. For each K value, 20 runs were carried out (100,000 burn-in generations, 200,000 Markov chain generations). The most likely K was determined following Evanno et al. [47] and Kopelman et al. [48]. All of the statistical analyses were carried out using the JMP version 7 software (SAS Institute Inc. 2007, Cary, NC, USA).

## Figures and Tables

**Table 1:** Flowering traits and origin of analyzed cultivars. The names of reference cultivars are preceded by an asterisk. (The capital letters represent Tukey–Kramer (TK) groups at *p* < 0.05. Cultivars sharing at least one TK symbol are not significantly different for the trait in the column).

Cultivar	Origin	Initial Flowering	Maximum Flowering	Final Flowering
*Antioco Pala*	Sardinia	$41.67{\;}^{C–I}$	$47.33{\;}^{C–J}$	$60.67{\;}^{A–C}$
*Antoni Piras*	Sardinia	$43.33{\;}^{B–H}$	$51.00{\;}^{A–H}$	$56.00{\;}^{A–C}$
*Arrubia*	Sardinia	$50.67{\;}^{A–D}$	$58.00{\;}^{A–C}$	$70.30{\;}^{A–C}$
*Basibi*	Sardinia	$35.67{\;}^{H–K}$	$42.67{\;}^{G–L}$	$62.00{\;}^{A–C}$
*Bianca*	Sardinia	$48.33{\;}^{A–G}$	$53.00{\;}^{A–G}$	$69.33{\;}^{A–C}$
*Bocchino*	Sardinia	$30.33{\;}^{I–L}$	$35.33{\;}^{LM}$	$52.00{\;}^{A–C}$
*Ciatta Inglese*	Sardinia	$40.00{\;}^{C–K}$	46.00 ${}^{D–L}$	$62.67{\;}^{A–C}$
*Ciatta Malissa*	Sardinia	$38.67{\;}^{D–K}$	45.67 ${}^{E–L}$	$60.33{\;}^{A–C}$
*Corrochina*	Sardinia	$33.33{\;}^{H–K}$	$43.00{\;}^{G–L}$	$61.00{\;}^{A–C}$
*Cossu*	Sardinia	$50.33{\;}^{A–E}$	$58.67{\;}^{AB}$	$72.33{\;}^{AB}$
*De Efisi Sinzoba*	Sardinia	$38.67{\;}^{D–K}$	$46.33{\;}^{D–K}$	$64.67{\;}^{A–C}$
*De Mrasciai*	Sardinia	$50.67{\;}^{A–D}$	$56.67{\;}^{A–D}$	$66.00{\;}^{A–C}$
*Efisi Sinzoba*	Sardinia	$29.67{\;}^{I–L}$	$38.33{\;}^{J–M}$	$62.00{\;}^{A–C}$
*Emilio 91*	Sardinia	$31.67{\;}^{H–K}$	$36.00{\;}^{K–M}$	$51.67{\;}^{BC}$
*Farci*	Sardinia	$38.00{\;}^{F–K}$	$44.67{\;}^{E–L}$	$59.67{\;}^{A–C}$
*Farrau*	Sardinia	$35.67{\;}^{H–K}$	$51.33{\;}^{A–H}$	$57.67{\;}^{A–C}$
*Fiori*	Sardinia	$32.00{\;}^{H–K}$	$41.33{\;}^{H–M}$	$59.00{\;}^{A–C}$
*Folla e pressiu*	Sardinia	33.33 ${}^{H–K}$	$39.33{\;}^{I–M}$	$60.33{\;}^{A–C}$
*Franciscu*	Sardinia	$39.33{\;}^{C–K}$	$45.00{\;}^{E–L}$	$62.33{\;}^{A–C}$
* Genco	Apulia	$55.67{\;}^{A}$	$60.67{\;}^{A}$	$70.67{\;}^{A–C}$
*Ghironi*	Sardinia	$42.67{\;}^{B–H}$	$49.33{\;}^{B–I}$	$62.33{\;}^{A–C}$
*Ibba*	Sardinia	$28.67{\;}^{J–L}$	$37.00{\;}^{J–M}$	$54.33{\;}^{A–C}$
*Is Stumbus*	Sardinia	$37.00{\;}^{G–K}$	$43.00{\;}^{G–L}$	$61.67{\;}^{A–C}$
*Lutzeddu*	Sardinia	$31.33{\;}^{H–K}$	$38.00{\;}^{J–M}$	$54.00{\;}^{A–C}$
*Malissa Tunda*	Sardinia	$49.00{\;}^{A–G}$	$54.00{\;}^{A–F}$	$65.00{\;}^{A–C}$
* Ne Plus Ultra	USA	$36.00{\;}^{H–K}$	$40.67{\;}^{H–M}$	$61.67{\;}^{A–C}$
*Niedda I*	Sardinia	$40.33{\;}^{C–J}$	$46.67{\;}^{D–K}$	$55.00{\;}^{A–C}$
*Niedda II*	Sardinia	$40.67{\;}^{C–J}$	$46.33{\;}^{D–K}$	$70.00{\;}^{A–C}$
* Nonpareil	USA	$49.67{\;}^{A–F}$	$58.33{\;}^{AB}$	$70.33{\;}^{A–C}$
*Nuxedda*	Sardinia	$40.33{\;}^{C–J}$	$45.00{\;}^{E–L}$	$64.00{\;}^{A–C}$
*Olla*	Sardinia	$28.00{\;}^{KL}$	$38.33{\;}^{J–M}$	$60.33{\;}^{A–C}$
*Orri*	Sardinia	37.67 ${}^{F–K}$	42.33 ${}^{G–M}$	$55.00{\;}^{A–C}$
* Picantili	Russia	$54.00{\;}^{AB}$	$60.33{\;}^{A}$	$71.33{\;}^{A–C}$
*Pitichedda*	Sardinia	$32.00{\;}^{H–K}$	$40.00{\;}^{I–M}$	$53.00{\;}^{A–C}$
*Provvista*	Sardinia	$31.33{\;}^{H–K}$	$37.67{\;}^{J–M}$	$58.67{\;}^{A–C}$
*Riu Loi*	Sardinia	$39.67{\;}^{C–K}$	$44.00{\;}^{F–L}$	$61.67{\;}^{A–C}$
*Schina de porcu*	Sardinia	$38.33{\;}^{E–K}$	$45.33{\;}^{E–L}$	$62.00{\;}^{A–C}$
*Stampasaccusu*	Sardinia	$18.67{\;}^{L}$	$31.67{\;}^{M}$	$50.67{\;}^{C}$
*Sunda G.*	Sardinia	$51.33{\;}^{A–C}$	$58.33{\;}^{AB}$	$69.00{\;}^{A–C}$
*Sunda N.*	Sardinia	$55.67{\;}^{A}$	61.33 ${}^{A}$	$72.33{\;}^{AB}$
* Troito A	Italy−Greece	$55.67{\;}^{A}$	$60.33{\;}^{A}$	$70.00{\;}^{A–C}$
* Troito B	Italy−Greece	$49.33{\;}^{A–F}$	$55.00{\;}^{A–E}$	$70.00{\;}^{A–C}$
* Tuono	Sardinia	$54.00{\;}^{AB}$	$60.00{\;}^{AB}$	$72.67{\;}^{A}$
*Vargiu*	Sardinia	$40.33{\;}^{C–J}$	$46.33{\;}^{D–K}$	$61.00{\;}^{A–C}$
*Vavani Perra*	Sardinia	$28.67{\;}^{J–L}$	$38.33{\;}^{J–M}$	$63.00{\;}^{A–C}$

**Table 2:** Analysis of variance of flowering, nut- and kernel- related traits. *** corresponds to *p* < 0.001, ** to *p* < 0.01, *ns* to *p* > 0.05.

**Initial Flowering**	**Factor**	**DF**	**SS**	**F**	**Max Flowering**	**Factor**	**DF**	**SS**	**F**
days after 1 Jan	Cultivar	44	10,543.08	$17.67{\;}^{***}$	days after 1 Jan	Cultivar	44	8943.06	$19.98{\;}^{***}$
	Year	2	759.53	$28.04{\;}^{***}$		Year	2	96.13	$4.70{\;}^{*}$
	Error	44	1191.81			Error	88	899.20	
**Final Flowering**	**Factor**	**DF**	**SS**	**F**	**Nut Weigth (gr)**	**Factor**	**DF**	**SS**	**F**
days after 1 jan	Cultivar	44	5031.08	$2.9{\;}^{**}$		Cultivar	44	286.06	$8.12{\;}^{***}$
	Year	2	477.57	$6.12{\;}^{**}$		Year	2	1.04	$0.62{\;}^{ns}$
	Error	88	3426.42			Error	88	70.38	
**Kernel Weight (g)**	**Factor**	**DF**	**SS**	**F**	**Kernel Shelling %**	**Factor**	**DF**	**SS**	**F**
	Cultivar	44	7.56	$5.99{\;}^{***}$		Cultivar	44	20,837.27	$81.82{\;}^{***}$
	Year	2	0.77	$13.48{\;}^{***}$		Year	2	122.29	$10.56{\;}^{***}$
	Error	88	2.52			Error	88	509.31	
**Kernel Yield** **(kg/plant)**	**Factor**	**DF**	**SS**	**F**	**Double Kernels %**	**Factor**	**DF**	**SS**	**F**
	Cultivar	44	72,155.65	$12.63{\;}^{***}$		Cultivar	44	20,777.78	$8.78{\;}^{***}$
	Year	2	30.706	$0.11{\;}^{ns}$		Year	2	834.311	$7.76{\;}^{ns}$
	Error	88	11,419.6			Error	88	4729.69	
**Failed Kernels %**	**Factor**	**DF**	**SS**	**F**	**Nut Length (cm)**	**Factor**	**DF**	**SS**	**F**
	Cultivar	44	557.34	$4.63{\;}^{***}$		Cultivar	44	13.30	$4.49{\;}^{***}$
	Year	2	13.38	$2.44{\;}^{ns}$		Year	2	0.12	$0.92{\;}^{ns}$
	Error	88	240.62			Error	88	5.92	
**Nut Width (cm)**	**Factor**	**DF**	**SS**	**F**	**Kernel Length (cm)**	**Factor**	**DF**	**SS**	**F**
	**Cultivar**	44	10.23	$2.77{\;}^{***}$		Cultivar	44	6.84	$9.25{\;}^{***}$
	Year	2	0.106	$0.63{\;}^{ns}$		Year	2	0.78	$2.33{\;}^{ns}$
	Error	88	7.32			Error	88	1.47	
**Kernel Width (cm)**	**Factor**	**DF**	**SS**	**F**					
	Cultivar	44	2.59	$6.93{\;}^{**}$					
	Year	2	0.03	$1.8{\;}^{ns}$					
	Error	88	0.74						

**Table 3:** Phenotypic traits of the almond cultivars. The names of the reference cultivars are preceded by an asterisk. (The capital letters represent Tukey–Kramer groups at *p* < 0.05. Cultivars sharing at least one TK symbol are not significantly different for the trait in the column).

Cultivar Name	Nut Weight (g)	Kernel Weight (g)	Kernel Yield (kg/plant)	Nut Length (cm)	Nut Width (cm)	Kernel Length (cm)	Kernel Width (cm)	Kernel Failed (%)	Kernel Double (%)
*Antioco Pala*	$5.56{\;}^{A–I}$	$1.53{\;}^{A–H}$	$0.77{\;}^{B}$	$3.02{\;}^{B–G}$	$2.44{\;}^{A–C}$	$2.52{\;}^{A–F}$	$1.53{\;}^{A–E}$	$1.00{\;}^{BC}$	$19.33{\;}^{B–H}$
*Antoni Piras*	$6.04{\;}^{A–H}$	$1.36{\;}^{A–I}$	$0.76{\;}^{B}$	$3.27{\;}^{A–G}$	$2.33{\;}^{BC}$	$2.29{\;}^{C–J}$	$1.44{\;}^{A–H}$	$1.67{\;}^{BC}$	$6.00{\;}^{F–H}$
*Arrubia*	$6.34{\;}^{A–G}$	$1.50{\;}^{A–H}$	$0.84{\;}^{B}$	$3.16{\;}^{B–G}$	$2.56{\;}^{A–C}$	$2.51{\;}^{A–F}$	$1.58{\;}^{A–D}$	$1.00{\;}^{BC}$	$1.00{\;}^{H}$
*Basibi*	$7.47{\;}^{A}$	$1.50{\;}^{A–H}$	$0.93{\;}^{B}$	$3.30{\;}^{A–F}$	$2.67{\;}^{A–C}$	$2.45{\;}^{B–I}$	$1.67{\;}^{A}$	$1.33{\;}^{BC}$	$0.67{\;}^{H}$
*Bianca*	$7.04{\;}^{A–D}$	$1.84{\;}^{A}$	$0.72{\;}^{B}$	$3.26{\;}^{A–G}$	$2.55{\;}^{A–C}$	$2.40{\;}^{B–J}$	$1.57{\;}^{A–E}$	$0.33{\;}^{BC}$	$43.33{\;}^{AB}$
*Bocchino*	$6.42{\;}^{A–F}$	$1.62{\;}^{A–E}$	$0.86{\;}^{B}$	$3.14{\;}^{B–G}$	$2.47{\;}^{A–C}$	$2.48{\;}^{A–H}$	$1.64{\;}^{AB}$	$2.33{\;}^{BC}$	$4.33{\;}^{F–H}$
*Ciatta Inglese*	$5.37{\;}^{A–I}$	$1.60{\;}^{A–E}$	$0.71{\;}^{B}$	$3.27{\;}^{A–G}$	$2.53{\;}^{A–C}$	$2.42{\;}^{B–J}$	$1.55{\;}^{A–E}$	$2.00{\;}^{BC}$	$7.00{\;}^{E–H}$
*Ciatta Malissa*	$7.09{\;}^{ABC}$	$1.57{\;}^{A–F}$	$0.67{\;}^{B}$	$3.09{\;}^{B–G}$	$2.81{\;}^{AB}$	$2.25{\;}^{C–J}$	$1.66{\;}^{A}$	$1.00{\;}^{BC}$	$11.33{\;}^{D–H}$
*Corrochina*	$4.96{\;}^{A–J}$	$1.41{\;}^{A–H}$	$0.62{\;}^{B}$	$3.34{\;}^{A–F}$	$2.66{\;}^{A–C}$	$2.68{\;}^{A–C}$	$1.38{\;}^{A–I}$	$1.00{\;}^{BC}$	$0.67{\;}^{H}$
*Cossu*	$6.32{\;}^{A–G}$	$1.74{\;}^{A–C}$	$1.05{\;}^{B}$	$3.33{\;}^{A–F}$	$2.67{\;}^{A–C}$	$2.37{\;}^{B–J}$	$1.67{\;}^{A}$	$2.33{\;}^{BC}$	$8.67{\;}^{E–H}$
*De Efisi Sinzoba*	$2.29{\;}^{JK}$	$1.43{\;}^{A–H}$	$0.81{\;}^{B}$	$3.18{\;}^{B–G}$	$1.83{\;}^{C}$	$2.48{\;}^{A–H}$	$1.19{\;}^{HI}$	$0.33{\;}^{BC}$	$15.67{\;}^{C–H}$
*De Mrasciai*	$6.23{\;}^{A–G}$	$1.27{\;}^{B–I}$	$0.62{\;}^{B}$	$2.89{\;}^{B–G}$	$2.75{\;}^{A–C}$	$2.12{\;}^{E–K}$	$1.48{\;}^{A–H}$	$0.00{\;}^{C}$	$0.00{\;}^{H}$
*Efisi Sinzoba*	$4.74{\;}^{A–J}$	$1.21{\;}^{B–I}$	$0.68{\;}^{B}$	$2.79{\;}^{C–G}$	$2.16{\;}^{BC}$	$2.07{\;}^{G–K}$	$1.34{\;}^{C–I}$	$0.33{\;}^{BC}$	$11.00{\;}^{D–H}$
*Emilio 91*	$6.73{\;}^{A–E}$	$1.73{\;}^{A–D}$	$0.65{\;}^{B}$	$3.26{\;}^{A–G}$	$2.58{\;}^{A–C}$	$2.44{\;}^{B–I}$	$1.61{\;}^{A–C}$	$0.67{\;}^{BC}$	$5.00{\;}^{F–H}$
*Farci*	$4.52{\;}^{A–J}$	$1.00{\;}^{F–I}$	$0.30{\;}^{B}$	$3.21{\;}^{A–G}$	$2.28{\;}^{BC}$	$2.24{\;}^{D–J}$	$1.29{\;}^{D–I}$	$2.00{\;}^{BC}$	$0.67{\;}^{H}$
*Farrau*	$6.41{\;}^{A–F}$	$1.54{\;}^{A–G}$	$0.90{\;}^{B}$	$3.34{\;}^{A–F}$	$2.48{\;}^{A–C}$	$2.61{\;}^{A–D}$	$1.54{\;}^{A–E}$	$0.33{\;}^{BC}$	$0.00{\;}^{H}$
*Fiori*	$3.39{\;}^{G–K}$	$1.74{\;}^{A–C}$	$0.67{\;}^{B}$	$3.29{\;}^{A–G}$	$2.35{\;}^{BC}$	$2.50{\;}^{A–G}$	$1.50{\;}^{A–F}$	$0.33{\;}^{BC}$	$5.00{\;}^{F–H}$
*Folla e pressiu*	$6.57{\;}^{A–F}$	$1.67{\;}^{A–E}$	$0.52{\;}^{B}$	$3.31{\;}^{A–F}$	$2.62{\;}^{A–C}$	$2.38{\;}^{B–J}$	$1.63{\;}^{A–C}$	$1.67{\;}^{B–C}$	$9.00{\;}^{E–H}$
*Franciscu*	$3.62{\;}^{F–K}$	$0.96{\;}^{HI}$	$0.28{\;}^{B}$	$2.87{\;}^{B–G}$	$1.95{\;}^{BC}$	$2.13{\;}^{E–K}$	$1.11{\;}^{I}$	$0.33{\;}^{BC}$	$7.33{\;}^{E–H}$
* Genco	$4.04{\;}^{E–K}$	$1.39{\;}^{A–I}$	$0.63{\;}^{B}$	$2.95{\;}^{B–G}$	$2.17{\;}^{BC}$	$2.29{\;}^{C–J}$	$1.42{\;}^{A–H}$	$0.67{\;}^{BC}$	$2.00{\;}^{H}$
*Ghironi*	$7.21{\;}^{A–C}$	$1.65{\;}^{A–E}$	$0.70{\;}^{B}$	$3.35{\;}^{A–F}$	$2.71{\;}^{A–C}$	$2.47{\;}^{A–H}$	$1.67{\;}^{A}$	$0.67{\;}^{BC}$	$34.33{\;}^{A–D}$
*Ibba*	$4.29{\;}^{B–K}$	$1.51{\;}^{A–H}$	$0.37{\;}^{B}$	$2.97{\;}^{B–G}$	$2.24{\;}^{BC}$	$2.39{\;}^{B–J}$	$1.28{\;}^{D–I}$	$1.67{\;}^{BC}$	$36.33{\;}^{ABC}$
*Is Stumbus*	$5.62{\;}^{A–I}$	$1.54{\;}^{A–G}$	$0.88{\;}^{B}$	$3.58{\;}^{A–D}$	$2.43{\;}^{A–C}$	$2.75{\;}^{AB}$	$1.44{\;}^{A–H}$	$0.00{\;}^{C}$	$0.33{\;}^{H}$
*Lutzeddu*	$7.27{\;}^{AB}$	$1.76{\;}^{AB}$	$0.58{\;}^{B}$	$3.55{\;}^{A–E}$	$2.55{\;}^{A–C}$	$2.50{\;}^{A–G}$	$1.51{\;}^{A–F}$	$2.00{\;}^{BC}$	$21.00{\;}^{B–H}$
*Malissa Tunda*	$6.67{\;}^{A–E}$	$1.33{\;}^{A–I}$	$0.19{\;}^{B}$	$3.73{\;}^{AB}$	$2.55{\;}^{A–C}$	$2.62{\;}^{A–D}$	$1.55{\;}^{A–E}$	$1.00{\;}^{BC}$	$3.33{\;}^{H}$
* Ne Plus Ultra	$2.76{\;}^{I–K}$	$1.66{\;}^{A–E}$	$0.99{\;}^{B}$	$4.05{\;}^{A}$	$2.22{\;}^{BC}$	$2.89{\;}^{A}$	$1.33{\;}^{C–I}$	$1.00{\;}^{BC}$	$22.67{\;}^{B–H}$
*Niedda I*	$7.28{\;}^{AB}$	$1.73{\;}^{A–E}$	$0.53{\;}^{B}$	$3.52{\;}^{A–E}$	$3.34{\;}^{A}$	$2.53{\;}^{A–F}$	$1.65{\;}^{AB}$	$0.67{\;}^{B–C}$	$22.67{\;}^{B–H}$
*Niedda II*	$6.22{\;}^{A–G}$	$1.31{\;}^{A–I}$	$1.02{\;}^{B}$	$3.17{\;}^{B–G}$	$2.35{\;}^{BC}$	$2.31{\;}^{C–J}$	$1.45{\;}^{A–H}$	$1.00{\;}^{BC}$	$2.00{\;}^{H}$
* Nonpareil	$1.33{\;}^{K}$	$0.99{\;}^{G–I}$	$0.11{\;}^{B}$	$3.16{\;}^{B–G}$	$1.80{\;}^{C}$	$2.42{\;}^{B–I}$	$1.22{\;}^{F–I}$	$13.00{\;}^{A}$	$0.67{\;}^{H}$
*Nuxedda*	$4.37{\;}^{B–J}$	$0.84{\;}^{I}$	$0.84{\;}^{B}$	$2.43{\;}^{G}$	$2.22{\;}^{BC}$	$1.74{\;}^{K}$	$1.28{\;}^{E–I}$	$1.67{\;}^{BC}$	$0.00{\;}^{H}$
*Olla*	$4.71{\;}^{A–J}$	$1.17{\;}^{E–I}$	$1.03{\;}^{B}$	$2.71{\;}^{E–G}$	$2.18{\;}^{BC}$	$2.02{\;}^{I–K}$	$1.33{\;}^{C–I}$	$0.33{\;}^{BC}$	$14.00{\;}^{C–H}$
*Orri*	$4.73{\;}^{A–J}$	$1.32{\;}^{A–I}$	$0.33{\;}^{B}$	$2.64{\;}^{FG}$	$2.28{\;}^{B–C}$	$2.05{\;}^{H–K}$	$1.41{\;}^{A–I}$	$8.50{\;}^{B}$	$28.33{\;}^{A–F}$
* Picantili	$2.84{\;}^{I–K}$	$1.59{\;}^{A–E}$	$0.47{\;}^{B}$	$3.36{\;}^{A–F}$	$2.32{\;}^{BC}$	$2.48{\;}^{A–H}$	$1.52{\;}^{A–F}$	$2.67{\;}^{BC}$	$9.67{\;}^{E–H}$
*Pitichedda*	$3.23{\;}^{H–K}$	$1.62{\;}^{A–E}$	$0.55{\;}^{B}$	$2.59{\;}^{FG}$	$2.24{\;}^{BC}$	$1.99{\;}^{JK}$	$1.43{\;}^{A–H}$	$0.00{\;}^{C}$	$48.33{\;}^{A}$
*Provvista*	$6.35{\;}^{A–G}$	$1.59{\;}^{A–E}$	$0.61{\;}^{B}$	$3.65{\;}^{AB}$	$2.50{\;}^{A–C}$	$2.59{\;}^{A–D}$	$1.53{\;}^{A–E}$	$2.33{\;}^{BC}$	$28.00{\;}^{A–G}$
*Riu Loi*	$4.67{\;}^{A–J}$	$1.19{\;}^{C–I}$	$0.75{\;}^{B}$	$2.90{\;}^{B–G}$	$2.36{\;}^{BC}$	$2.06{\;}^{H–K}$	$1.47{\;}^{A–H}$	$1.00{\;}^{BC}$	$0.00{\;}^{H}$
*Schina de porcu*	$4.47{\;}^{B–J}$	$1.56{\;}^{A–F}$	$0.67{\;}^{B}$	$3.23{\;}^{A–G}$	$2.53{\;}^{A–C}$	$2.43{\;}^{B–I}$	$1.58{\;}^{A–E}$	$0.33{\;}^{BC}$	$5.33{\;}^{F–H}$
*Stampasaccusu*	$5.92{\;}^{A–H}$	$1.23{\;}^{B–I}$	$0.75{\;}^{B}$	$3.32{\;}^{A–F}$	$2.16{\;}^{BC}$	$2.56{\;}^{A–E}$	$1.20{\;}^{G–I}$	$1.00{\;}^{BC}$	$10.67{\;}^{D–H}$
*Sunda G.*	$6.21{\;}^{A–H}$	$1.34{\;}^{A–I}$	$0.54{\;}^{B}$	$3.38{\;}^{A–F}$	$2.89{\;}^{AB}$	$2.40{\;}^{B–J}$	$1.60{\;}^{A–C}$	$1.00{\;}^{BC}$	$3.67{\;}^{GH}$
*Sunda N.*	$5.19{\;}^{A–J}$	$1.21{\;}^{B–I}$	$0.54{\;}^{B}$	$3.10{\;}^{B–G}$	$2.24{\;}^{BC}$	$2.35{\;}^{B–J}$	$1.35{\;}^{B–I}$	$2.67{\;}^{BC}$	$7.00{\;}^{E–H}$
* Troito A	$6.32{\;}^{A–G}$	$1.22{\;}^{B–I}$	$1.22{\;}^{A–B}$	$3.35{\;}^{A–F}$	$2.57{\;}^{A–C}$	$2.33{\;}^{B–J}$	$1.51{\;}^{A–F}$	$0.67{\;}^{BC}$	$4.67{\;}^{F–H}$
* Troito B	$4.07{\;}^{D–K}$	$1.29{\;}^{A–I}$	$0.61{\;}^{B}$	$2.85{\;}^{C–G}$	$2.28{\;}^{BC}$	$2.11{\;}^{F–K}$	$1.38{\;}^{A–I}$	$1.00{\;}^{BC}$	$1.67{\;}^{H}$
* Tuono	$4.26{\;}^{C–K}$	$1.45{\;}^{A–H}$	$2.46{\;}^{A}$	$3.13{\;}^{B–G}$	$2.34{\;}^{BC}$	$2.34{\;}^{B–J}$	$1.49{\;}^{A–G}$	$0.00{\;}^{C}$	$30.67{\;}^{A–E}$
*Vargiu*	$4.96{\;}^{A–J}$	$1.17{\;}^{D–I}$	$1.11{\;}^{B}$	$2.79{\;}^{C–G}$	$2.24{\;}^{BC}$	$2.10{\;}^{F–K}$	$1.45{\;}^{A–H}$	$3.00{\;}^{BC}$	$1.67{\;}^{H}$
*Vavani Perra*	$4.91{\;}^{A–J}$	$1.19{\;}^{C–I}$	$1.19{\;}^{B}$	$2.72{\;}^{DEFG}$	$2.11{\;}^{BC}$	$2.03{\;}^{I–K}$	$1.35{\;}^{B–I}$	$0.00{\;}^{C}$	$8.00{\;}^{E–H}$

**Table 4:** Analysis of variance for commercial traits of the almond cultivars. *** corresponds to *p* < 0.001. C × Y means the interaction Cultivar by Year.

**Trait**	**Source**	**DF**	**SS**	**F**	**Trait**	**Source**	**DF**	**SS**	**F-Value**
**Total oil**	Cultivar	44	3351.96	24.8634 ${}^{***}$	**Palmitic acid**	Cultivar	44	72.32	156.01 ${}^{***}$
	Year	2	139.73	6.1521 ${}^{***}$		Year	2	6.39	304.69 ${}^{***}$
	C × Y	88	1658.78	6.1521 ${}^{***}$		C × Y	88	11.79	12.77 ${}^{***}$
	Error	270	827.27			Error	270	2.83	
**Trait**	**Source**	**DF**	**SS**	**F**	**Trait**	**Source**	**DF**	**SS**	**F**
**Palmitoleic acid**	Cultivar	44	2.61	76.74 ${}^{***}$	**Stearic acid**	Cultivar	44	25.18	96.92 ${}^{***}$
	Year	2	1.67	1077.08 ${}^{***}$		Year	2	2.87	243.47 ${}^{***}$
	C × Y	88	8.76	12.77 ${}^{***}$		C × Y	88	6.44	12.40 ${}^{***}$
	Error	270	0.20			Error	270	1.59	
**Trait**	**Source**	**DF**	**SS**	**F**	**Trait**	**Source**	**DF**	**SS**	**F**
**Oleic acid**	Cultivar	44	3669.07	241.95 ${}^{***}$	**Linoleic acid**	Cultivar	44	2928.90	244.40 ${}^{***}$
	Year	2	262.96	381.51 ${}^{***}$		Year	2	295.56	542.59 ${}^{***}$
	C × Y	88	1078.70	35.57 ${}^{***}$		C × Y	88	877.18	36.60 ${}^{***}$
	Error	270	93.05			Error	270	73.53	
**Trait**	**Source**	**DF**	**SS**	**F**	**Trait**	**Source**	**DF**	**SS**	**F**
**Linolenic acid**	Cultivar	44	1534,850.9	126.1863 ${}^{***}$	**Tocopherol acid**	Cultivar	44	0.008	22.32 ${}^{***}$
	Year	2	870,549.8	1574.571 ${}^{***}$		Year	2	0.015	849.06 ${}^{***}$
	C × Y	88	363,388.7	14.9378 ${}^{***}$		C × Y	88	0.013	17.22 ${}^{***}$
	Error	270	8.63e-6			Error	270	74.36	

**Table 5:** Oil traits. The names of reference cultivars are preceded by an asterisk. (The superscript symbols after means represent Tukey–Kramer groups at *p* < 0.05. Cultivars sharing at least one TK symbol are not significantly different for the trait in the column).

Cultivar Name	Oil (% on d.w.)	Palmitic (% of Oil)	Palmitoleic (% of Oil)	Stearic (% of Oil)	Oleic (% of Oil)	Linoleic (% of Oil)	Tocopherol (mg/kg of Oil)
*Antioco Pala*	$54.74{\;}^{P–T}$	$5.87{\;}^{V–Z}$	$0.52{\;}^{L–P}$	$1.82{\;}^{J–O}$	$75.49{\;}^{D}$	$16.12{\;}^{ST}$	$345.43{\;}^{R–U}$
*Antoni Piras*	$58.50{\;}^{F–N}$	$6.78{\;}^{B–D}$	$0.57{\;}^{E–K}$	$2.21{\;}^{B–D}$	$70.61{\;}^{M–P}$	$19.61{\;}^{H–J}$	$423.10{\;}^{G–J}$
*Arrubia*	$55.01{\;}^{O–T}$	$6.73{\;}^{B–E}$	$0.66{\;}^{BC}$	$1.64{\;}^{R–T}$	$71.69{\;}^{K–M}$	$19.08{\;}^{J–L}$	$337.83{\;}^{TU}$
*Basibi*	$61.68{\;}^{A–F}$	$6.09{\;}^{N–T}$	$0.42{\;}^{T–V}$	$2.12{\;}^{C–F}$	$73.17{\;}^{G–I}$	$17.92{\;}^{N–P}$	$415.23{\;}^{H–M}$
*Bianca*	$62.37{\;}^{A–C}$	$5.85{\;}^{W–Z}$	$0.45{\;}^{R–U}$	$2.17{\;}^{B–D}$	$73.22{\;}^{F–I}$	$18.09{\;}^{M–P}$	$460.03{\;}^{C–F}$
*Bocchino*	$57.68{\;}^{I–P}$	$6.91{\;}^{B}$	$0.59{\;}^{E–I}$	$1.85{\;}^{I–N}$	$69.84{\;}^{P}$	$20.62{\;}^{D–G}$	$329.79{\;}^{UV}$
*Ciatta Inglese*	$57.88{\;}^{H–P}$	$6.19{\;}^{L–R}$	$0.77{\;}^{A}$	$1.62{\;}^{ST}$	$75.29{\;}^{DE}$	$15.93{\;}^{S–U}$	$375.83{\;}^{O–R}$
*Ciatta Malissa*	$59.38{\;}^{C–J}$	$6.38{\;}^{I–L}$	$0.64{\;}^{B–D}$	$1.90{\;}^{H–L}$	$71.88{\;}^{J–L}$	$18.98{\;}^{J–M}$	$404.50{\;}^{I–O}$
*Corrochina*	$60.44{\;}^{B–I}$	$6.64{\;}^{C–F}$	$0.59{\;}^{D–H}$	$1.72{\;}^{M–S}$	$71.06{\;}^{L–O}$	$19.80{\;}^{G–J}$	$472.19{\;}^{C–E}$
*Cossu*	$57.56{\;}^{I–Q}$	$6.01{\;}^{R–W}$	$0.49{\;}^{N–R}$	$2.42{\;}^{A}$	$73.99{\;}^{FG}$	$16.86{\;}^{Q–S}$	$389.87{\;}^{K–P}$
*De Efisi Sinzoba*	$60.36{\;}^{B–I}$	$6.65{\;}^{C–E}$	$0.57{\;}^{F–L}$	$1.74{\;}^{M–S}$	$68.64{\;}^{Q}$	$22.22{\;}^{C}$	$387.51{\;}^{L–P}$
*De Mrasciai*	$58.10{\;}^{G–O}$	$6.23{\;}^{K–P}$	$0.43{\;}^{S–V}$	$1.46{\;}^{UV}$	$78.29{\;}^{B}$	$13.40{\;}^{XY}$	$370.09{\;}^{P–S}$
*Efisi Sinzoba*	$57.68{\;}^{I–P}$	$6.04{\;}^{P–V}$	$0.57{\;}^{G–M}$	$1.80{\;}^{K–P}$	$76.91{\;}^{C}$	$14.50{\;}^{VW}$	$428.39{\;}^{G–I}$
*Emilio91*	$56.88{\;}^{J–Q}$	$6.66{\;}^{C–E}$	$0.62{\;}^{C–G}$	$2.02{\;}^{E–H}$	$71.05{\;}^{L–O}$	$19.45{\;}^{I–H}$	$301.28{\;}^{V}$
*Farci*	$56.76{\;}^{J–Q}$	$7.28{\;}^{A}$	$0.62{\;}^{C–F}$	$1.40{\;}^{UV}$	$69.51{\;}^{PQ}$	$21.00{\;}^{DE}$	$589.41{\;}^{A}$
*Farrau*	$52.98{\;}^{R–T}$	$7.38{\;}^{A}$	$0.58{\;}^{E–J}$	$1.66{\;}^{P–T}$	$70.42{\;}^{N–P}$	$19.76{\;}^{G–J}$	$419.69{\;}^{G–K}$
*Fiori*	$61.31{\;}^{A–G}$	$6.62{\;}^{D–G}$	$0.53{\;}^{J–O}$	$1.76{\;}^{L–S}$	$69.61{\;}^{PQ}$	$21.30{\;}^{C–E}$	$464.99{\;}^{C–F}$
*Foll’e pressiu*	$56.06{\;}^{K–R}$	$6.29{\;}^{I–M}$	$0.50{\;}^{N–Q}$	$1.87{\;}^{I–M}$	$73.96{\;}^{FG}$	$17.17{\;}^{P–R}$	$341.96{\;}^{S–U}$
*Franciscu*	$58.89{\;}^{D–L}$	$6.18{\;}^{M–R}$	$0.47{\;}^{P–S}$	$1.84{\;}^{J–N}$	$72.80{\;}^{H–J}$	$18.51{\;}^{K–N}$	$370.78{\;}^{P–S}$
* Genco	$60.65{\;}^{B–I}$	$5.28{\;}^{A}$	$0.54{\;}^{I–N}$	$1.53{\;}^{TU}$	$79.66{\;}^{A}$	$12.83{\;}^{Y}$	$226.90{\;}^{W}$
*Ghironi*	$63.33{\;}^{AB}$	$5.85{\;}^{W–Z}$	$0.47{\;}^{P–S}$	$2.19{\;}^{B–D}$	$73.91{\;}^{FG}$	$17.37{\;}^{PQ}$	$487.06{\;}^{C}$
*Ibba*	$64.47{\;}^{A}$	$6.42{\;}^{H–K}$	$0.48{\;}^{O–S}$	$2.14{\;}^{C–E}$	$69.89{\;}^{P}$	$20.86{\;}^{D–F}$	$382.31{\;}^{N–Q}$
*Is Stumbus*	$60.36{\;}^{B–I}$	$6.40{\;}^{H–K}$	$0.62{\;}^{C–G}$	$1.96{\;}^{G–J}$	$71.92{\;}^{J–L}$	$18.93{\;}^{J–M}$	$372.07{\;}^{P–S}$
*Lutzeddu*	$59.34{\;}^{C–K}$	$5.91{\;}^{T–Y}$	$0.52{\;}^{L–P}$	$1.79{\;}^{K–Q}$	$73.22{\;}^{F–H}$	$18.38{\;}^{L–O}$	$441.09{\;}^{E–H}$
*Malissa Tunda*	$52.03{\;}^{T}$	$5.88{\;}^{U–Y}$	$0.38{\;}^{V}$	$1.36{\;}^{V}$	$72.45{\;}^{H–K}$	$19.75{\;}^{G–J}$	$472.08{\;}^{C–E}$
* Ne Plus Ultra	$62.18{\;}^{A–D}$	$6.58{\;}^{E–H}$	$0.44{\;}^{S–U}$	$1.83{\;}^{J–O}$	$65.18{\;}^{S}$	$25.78{\;}^{A}$	$475.72{\;}^{CD}$
*Niedda I*	$55.68{\;}^{L–R}$	$5.97{\;}^{S–X}$	$0.54{\;}^{H–N}$	$1.78{\;}^{K–R}$	$74.32{\;}^{EF}$	$17.18{\;}^{P–R}$	$412.38{\;}^{H–N}$
*Niedda II*	$54.35{\;}^{Q–T}$	$5.79{\;}^{X–Z}$	$0.48{\;}^{P–S}$	$1.69{\;}^{O–S}$	$75.59{\;}^{D}$	$16.27{\;}^{R–T}$	$449.37{\;}^{D–G}$
* Non Pareil	$57.82{\;}^{H–P}$	$5.94{\;}^{T–Y}$	$0.51{\;}^{N–P}$	$1.65{\;}^{Q–T}$	$71.17{\;}^{L–O}$	$20.52{\;}^{E–H}$	$459.79{\;}^{C–F}$
*Nuxedda*	$55.51{\;}^{M–R}$	$6.21{\;}^{L–Q}$	$0.53{\;}^{K–O}$	$1.77{\;}^{K–S}$	$75.31{\;}^{DE}$	$15.98{\;}^{S–U}$	$417.50{\;}^{H–L}$
*Olla*	$57.79{\;}^{I–P}$	$6.03{\;}^{Q–W}$	$0.55{\;}^{H–N}$	$2.07{\;}^{D–G}$	$75.52{\;}^{D}$	$15.61{\;}^{TU}$	$416.81{\;}^{H–L}$
*Orri*	$61.10{\;}^{B–H}$	$5.96{\;}^{S–Y}$	$0.41{\;}^{UV}$	$1.85{\;}^{I–N}$	$76.03{\;}^{CD}$	$15.55{\;}^{TU}$	$383.40{\;}^{N–P}$
* Picantili	$52.14{\;}^{ST}$	$6.44{\;}^{G–J}$	$0.45{\;}^{R–U}$	$2.02{\;}^{E–H}$	$66.56{\;}^{R}$	$24.30{\;}^{B}$	$438.63{\;}^{F–H}$
*Pitichedda*	$59.50{\;}^{C–J}$	$6.26{\;}^{I–N}$	$0.47{\;}^{P–T}$	$1.82{\;}^{J–O}$	$73.34{\;}^{F–H}$	$17.92{\;}^{N–P}$	$364.22{\;}^{P–T}$
*Provvista*	$59.49{\;}^{C–J}$	5.68 ${}^{Z[}$	$0.52{\;}^{M–P}$	$1.91{\;}^{H–K}$	$74.32{\;}^{EF}$	$17.40{\;}^{O–Q}$	$486.33{\;}^{C}$
*Riu Loi*	$58.79{\;}^{E–M}$	$6.07{\;}^{O–U}$	$0.45{\;}^{R–U}$	$2.19{\;}^{B–D}$	$69.53{\;}^{PQ}$	$21.56{\;}^{CD}$	$390.14{\;}^{K–P}$
*Schina de Porcu*	$57.74{\;}^{I–P}$	$6.27{\;}^{I–N}$	$0.69{\;}^{B}$	$1.67{\;}^{P–T}$	$73.50{\;}^{F–H}$	$17.67{\;}^{N–Q}$	$351.28{\;}^{Q–U}$
*Stampasaccusu*	$54.88{\;}^{O–T}$	$6.82{\;}^{BC}$	$0.51{\;}^{N–P}$	$1.99{\;}^{F–I}$	$70.13{\;}^{OP}$	$20.35{\;}^{E–I}$	$392.67{\;}^{J–P}$
*Sunda G.*	$58.05{\;}^{G–O}$	$6.25{\;}^{J–O}$	$0.58{\;}^{E–K}$	$2.23{\;}^{BC}$	$72.12{\;}^{I–L}$	$18.61{\;}^{K–N}$	$438.52{\;}^{F–H}$
*Sunda N.*	$60.02{\;}^{C–J}$	$5.92{\;}^{T–Y}$	$0.50{\;}^{N–R}$	$2.02{\;}^{E–H}$	$71.47{\;}^{K–N}$	$19.89{\;}^{F–J}$	$463.59{\;}^{C–F}$
* TroitoA	$61.97{\;}^{A–E}$	$5.77{\;}^{Y–Z}$	$0.46{\;}^{Q–U}$	$1.69{\;}^{O–S}$	$78.05{\;}^{B}$	$13.84{\;}^{WX}$	$392.67{\;}^{J–P}$
* TroitoB	$62.07{\;}^{A–E}$	$6.45{\;}^{F–I}$	$0.54{\;}^{I–N}$	$2.30{\;}^{AB}$	$69.65{\;}^{PQ}$	$20.85{\;}^{D–F}$	$415.19{\;}^{H–M}$
* Tuono	$56.79{\;}^{J–Q}$	$6.15{\;}^{M–S}$	$0.42{\;}^{T–V}$	$2.43{\;}^{A}$	$73.45{\;}^{F–H}$	$17.32{\;}^{PQ}$	$540.84{\;}^{B}$
*Vargiu*	$57.97{\;}^{H–P}$	5.56 ${}^{[}$	$0.46{\;}^{Q–U}$	$1.85{\;}^{I–N}$	$76.92{\;}^{C}$	$15.01{\;}^{UV}$	$385.14{\;}^{M–P}$
*Vavani Perra*	$55.35{\;}^{N–S}$	$6.33{\;}^{I–M}$	$0.62{\;}^{C–E}$	$1.71{\;}^{N–S}$	$75.73{\;}^{D}$	$15.43{\;}^{T–V}$	$383.99{\;}^{M–P}$

**Table 6:** Genetic groups identified within the collection are differentiated according to phenotypic and quality-related traits. Data are means, and clusters’ differentiation was tested by the nonparametric Wilcoxon test. Only the variables for which Prob > Chi-square was below 0.05 are listed.

Trait	Variable	CL-A	CL-B
**Flowering**	**Start (days after 1 Jan)**	33.75	39.92
	**Maximum (days after the 1 Jan)**	38.67	47.13
	**Final (days after the 1 Jan)**	56.4	62.39
**Kernel**	**Width (cm)**	1.58	1.44
**Oil composition**	**Stearic (% of oil)**	1.98	1.81
	**Tocopherol (mg/kg)**	340.79	420.10

## Supplementary Materials

The following files are available online at http://www.mdpi.com/2223-7747/7/4/86/s1, Table S1: Additional detail on morphological, agronomic, and oil-related traits; Table S2: Eigenvalue and Eigenvectors of PCA; Table S3: Primer sequences and annealing temperatures. Supplemental file S1 reports additional information on cultivars used in genetic structure analysis and on best K determination.
