# Supplementary material for: Genetic, Phenotypic, and Commercial Characterization of an Almond Collection from Sardinia
Source: Plants (Basel). 2018 Oct 15;7(4):86. doi: 10.3390/plants7040086 (PMC6313889; doi:10.3390/plants7040086)
Supplement: Supplementary file 1 [file plants-07-00086-s001.zip › plants-361681-proofreading-supple/Supplemental_file_S1.docx]

**The file contains additional information that are mentioned in the main manuscript.**

1. Origin and identity of cultivars used in the study only for cluster analysis based on SSR data (see Figure 3 in the main manuscript).

| Rebeccu 1 | Sardinia |
| --- | --- |
| Rebeccu 2 | Sardinia |
| Rebeccu 3 | Sardinia |
| Jordanolo | USA/Commercial |
| Aldrich | USA/Commercial |
| Mission | USA/Commercial |
| Ruby | USA/Commercial |
| Sonora | USA/Commercial |
| Sweetheart | USA/Commercial |
| Winter | USA/Commercial |

2. Analysis of best K for the collection of Sardinian and reference USA cultivars as determined by the method of Evanno (see Figure 3 of the main text).


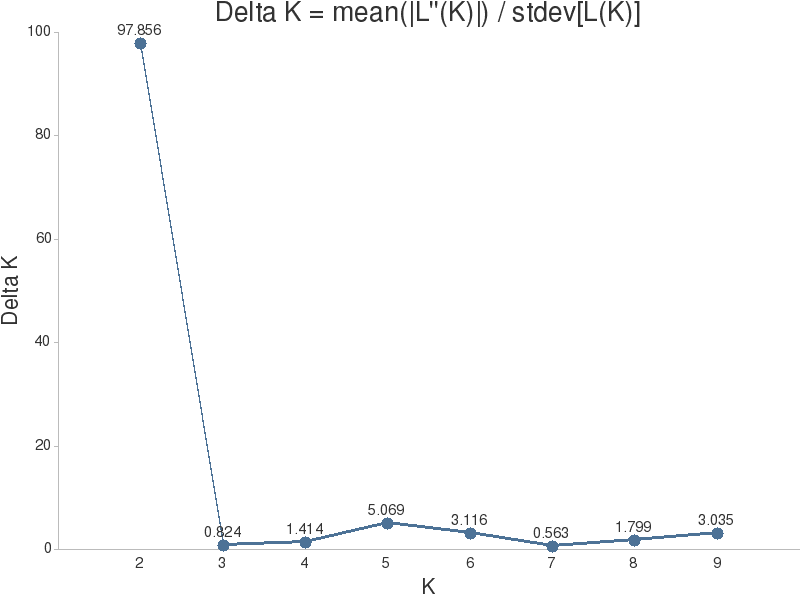


3. Analysis of the best K for the collection of Sardinian cultivars (see figure 4 of the main text).

**
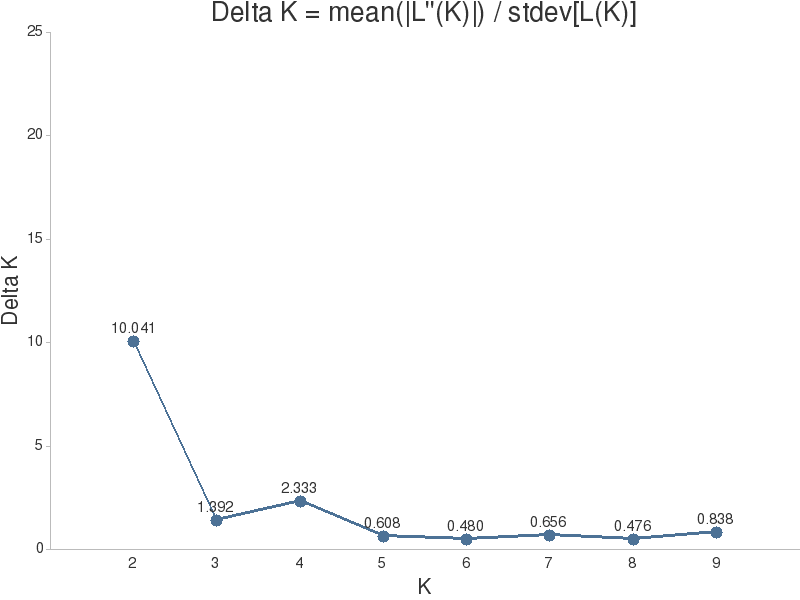
**
