# Supplementary material for: Genetic, Phenotypic, and Commercial Characterization of an Almond Collection from Sardinia
Source: Plants (Basel). 2018 Oct 15;7(4):86. doi: 10.3390/plants7040086 (PMC6313889; doi:10.3390/plants7040086)
Supplement: Supplementary file 1 [file plants-07-00086-s001.zip › plants-361681-proofreading-supple/Supplemental_Table_S2.docx]

Table S2

**Eigenvalues of PCA.**

Eigen values, percent of variance and cumulative variance explained by each dimension

|  | **eigenvalue** | **variance.percent** | **cumulative.variance.percent** |
| --- | --- | --- | --- |
| Dim.1 | 3.996 | 19.981 | 19.981 |
| Dim.2 | 3.816 | 19.081 | 39.062 |
| Dim.3 | 3.117 | 15.587 | 54.649 |
| Dim.4 | 2.052 | 10.261 | 64.910 |
| Dim.5 | 1.511 | 7.556 | 72.466 |
| Dim.6 | 1.283 | 6.413 | 78.879 |
| Dim.7 | 1.086 | 5.429 | 84.308 |
| Dim.8 | 0.766 | 3.831 | 88.139 |
| Dim.9 | 0.644 | 3.222 | 91.361 |
| Dim.10 | 0.604 | 3.019 | 94.380 |
| Dim.11 | 0.367 | 1.834 | 96.214 |
| Dim.12 | 0.247 | 1.236 | 97.450 |
| Dim.13 | 0.173 | 0.864 | 98.314 |
| Dim.14 | 0.129 | 0.645 | 98.959 |
| Dim.15 | 0.065 | 0.324 | 99.283 |
| Dim.16 | 0.053 | 0.266 | 99.549 |
| Dim.17 | 0.050 | 0.250 | 99.799 |
| Dim.18 | 0.027 | 0.136 | 99.935 |
| Dim.19 | 0.013 | 0.065 | 100.000 |
| Dim.20 | 0.000 | 0.000 | 100.000 |

**Loadings of variables in the PCA axes.**

| **Original variables** | **Dim.1** | **Dim.2** | **Dim.3** | **Dim.4** | **Dim.5** | **Dim.6** | **Dim.7** | **Dim.8** |
| --- | --- | --- | --- | --- | --- | --- | --- | --- |
| palmitic | 0.5390 | -0.3790 | -0.1683 | -0.3000 | 0.5033 | 0.0021 | -0.1462 | -0.2310 |
| palmitoleic | 0.2319 | -0.0158 | -0.2355 | -0.5579 | 0.3251 | 0.3951 | 0.2896 | 0.2238 |
| stearic_oleic | 0.0417 | 0.1814 | 0.2112 | 0.6537 | 0.4458 | -0.0871 | 0.2677 | -0.2281 |
| oleic | -0.6837 | 0.5682 | -0.2790 | -0.0297 | -0.2444 | 0.0713 | -0.0183 | 0.2065 |
| Linoleic | 0.6631 | -0.5911 | 0.3253 | 0.0358 | 0.1324 | -0.0832 | 0.0067 | -0.1764 |
| Linolenic | -0.0141 | -0.0257 | 0.4397 | -0.1715 | 0.1585 | -0.1296 | 0.7359 | 0.2850 |
| tocopherol | 0.1803 | -0.2097 | 0.3583 | 0.1736 | 0.1289 | -0.6816 | -0.1403 | 0.3021 |
| initial_flowering | -0.4489 | 0.2064 | 0.7919 | -0.1402 | 0.0040 | 0.0816 | -0.1347 | -0.1806 |
| max_flowering | -0.4262 | 0.1666 | 0.8013 | -0.1792 | 0.0529 | 0.0723 | -0.1389 | -0.1627 |
| final_flowering | -0.5369 | 0.0731 | 0.7401 | -0.1018 | 0.1508 | 0.1057 | -0.1204 | 0.1431 |
| nut_weight | 0.3008 | 0.8497 | -0.1218 | -0.1268 | 0.0341 | -0.2879 | 0.0252 | -0.0673 |
| Kernel_weight | 0.6702 | 0.4081 | 0.1610 | 0.3267 | -0.1465 | 0.4193 | 0.0533 | -0.0131 |
| shelling. | 0.0841 | -0.7036 | 0.3471 | 0.2196 | -0.2545 | 0.4391 | 0.0480 | 0.0320 |
| kernel_yield | -0.2207 | 0.1470 | 0.1229 | 0.4565 | 0.6168 | 0.2460 | -0.0925 | 0.2331 |
| double_kernels | 0.1505 | 0.0608 | -0.0585 | 0.8132 | -0.2596 | 0.0177 | 0.0863 | 0.0951 |
| nuts_with_kernel_failed | -0.1637 | -0.4002 | 0.2748 | -0.0969 | -0.4298 | -0.1909 | 0.4259 | -0.2360 |
| Nut_length | 0.7234 | 0.1402 | 0.4595 | -0.0909 | -0.1742 | -0.0614 | -0.2394 | 0.2422 |
| Nut_width | 0.3933 | 0.7989 | 0.1486 | -0.1240 | -0.0021 | -0.1187 | 0.0901 | -0.0702 |
| Kernel_length | 0.7693 | 0.0208 | 0.3650 | -0.1599 | -0.2407 | 0.1122 | -0.1067 | 0.2214 |
| Kernel_width | 0.3526 | 0.7871 | 0.2122 | -0.0205 | -0.0272 | 0.1527 | 0.1237 | -0.2179 |

| **Original variables** | **Dim.9** | **Dim.10** | **Dim.11** | **Dim.12** | **Dim.13** | **Dim.14** | **Dim.15** | **Dim.16** |
| --- | --- | --- | --- | --- | --- | --- | --- | --- |
| palmitic | 0.1647 | 0.1289 | 0.0055 | 0.2132 | 0.0321 | 0.1587 | -0.0051 | 0.0473 |
| palmitoleic | 0.1529 | 0.2252 | 0.2999 | -0.1096 | 0.0107 | -0.0826 | 0.0095 | -0.0032 |
| stearic_oleic | -0.2194 | -0.1228 | 0.2396 | -0.1428 | 0.1271 | 0.0749 | -0.0224 | -0.0002 |
| oleic | -0.0284 | 0.0919 | 0.0256 | 0.0348 | 0.0683 | 0.0759 | -0.0342 | 0.0061 |
| Linoleic | 0.0208 | -0.1207 | -0.0632 | -0.0574 | -0.0947 | -0.1197 | 0.0412 | -0.0144 |
| Linolenic | 0.0892 | -0.2521 | -0.1577 | 0.1079 | -0.0177 | 0.0239 | -0.0453 | 0.0174 |
| tocopherol | 0.1958 | 0.3385 | -0.0023 | -0.1149 | -0.0089 | 0.0377 | -0.0499 | -0.0507 |
| initial_flowering | 0.1357 | -0.0222 | 0.0699 | 0.0570 | 0.0201 | -0.1301 | -0.0165 | -0.0086 |
| max_flowering | 0.1520 | -0.0056 | 0.1013 | 0.1090 | 0.0590 | 0.0093 | -0.0791 | -0.0343 |
| final_flowering | -0.0032 | -0.0335 | 0.0455 | -0.1074 | -0.1538 | 0.1410 | 0.1380 | 0.0424 |
| nut_weight | -0.0898 | -0.0219 | 0.0969 | 0.1144 | -0.1460 | 0.0248 | -0.0117 | 0.0439 |
| Kernel_weight | 0.0797 | 0.0782 | -0.0135 | -0.0120 | -0.0919 | 0.0777 | -0.0205 | -0.0568 |
| shelling. | 0.0631 | 0.1194 | -0.1392 | -0.0619 | 0.0863 | 0.0810 | -0.0388 | 0.0556 |
| kernel_yield | -0.2770 | 0.2460 | -0.1605 | 0.1700 | -0.0056 | -0.0979 | 0.0206 | -0.0106 |
| double_kernels | 0.3917 | 0.0085 | 0.1843 | 0.1605 | -0.0387 | -0.0477 | 0.0479 | 0.0405 |
| nuts_with_kernel_failed | -0.2793 | 0.4113 | 0.0877 | 0.1078 | -0.0095 | -0.0027 | 0.0523 | 0.0048 |
| Nut_length | -0.2158 | -0.0232 | 0.0849 | -0.0149 | 0.0757 | -0.0584 | -0.0381 | 0.1425 |
| Nut_width | 0.1798 | 0.0683 | -0.1772 | -0.0288 | 0.2349 | -0.0140 | 0.1310 | -0.0043 |
| Kernel_length | -0.2262 | -0.1193 | 0.1070 | 0.1176 | 0.0402 | 0.0598 | 0.0238 | -0.1190 |
| Kernel_width | 0.0100 | 0.2202 | -0.1620 | -0.1232 | -0.1118 | -0.0083 | -0.0673 | 0.0154 |

| **Original variables** | **Dim.17** | **Dim.18** | **Dim.19** | **Dim.20** |
| --- | --- | --- | --- | --- |
| palmitic | 0.0203 | -0.0363 | 0.0134 | 0.0006 |
| palmitoleic | 0.0000 | 0.0188 | -0.0008 | 0.0001 |
| stearic_oleic | 0.0091 | -0.0011 | 0.0099 | 0.0004 |
| oleic | 0.0049 | -0.0164 | 0.0083 | 0.0042 |
| Linoleic | -0.0082 | 0.0193 | -0.0125 | 0.0038 |
| Linolenic | -0.0029 | -0.0182 | 0.0025 | 0.0000 |
| tocopherol | -0.0118 | 0.0030 | 0.0114 | 0.0000 |
| initial_flowering | -0.0188 | -0.0328 | 0.0742 | 0.0000 |
| max_flowering | -0.0081 | 0.0393 | -0.0684 | 0.0000 |
| final_flowering | 0.0153 | -0.0066 | -0.0056 | 0.0000 |
| nut_weight | -0.0483 | 0.0982 | 0.0239 | 0.0001 |
| Kernel_weight | -0.1351 | -0.0462 | -0.0051 | 0.0000 |
| shelling. | -0.0031 | 0.0857 | 0.0292 | 0.0001 |
| kernel_yield | -0.0025 | 0.0132 | -0.0048 | 0.0000 |
| double_kernels | 0.0612 | -0.0013 | -0.0072 | 0.0000 |
| nuts_with_kernel_failed | -0.0165 | -0.0162 | -0.0077 | 0.0000 |
| Nut_length | -0.0183 | -0.0386 | -0.0164 | 0.0000 |
| Nut_width | -0.0195 | 0.0176 | -0.0058 | 0.0000 |
| Kernel_length | 0.0840 | 0.0137 | 0.0177 | 0.0000 |
| Kernel_width | 0.1276 | -0.0167 | -0.0037 | 0.0000 |
